# Supplementary material for: Visual working memories are abstractions of percepts
Source: eLife. 2024 May 31;13:RP94191. doi: 10.7554/eLife.94191 (PMC11147505; doi:10.7554/eLife.94191)
Supplement: Supplementary file 1. — (a) Decoding accuracy for the stimulus-presenting epoch in the perceptual control task. (b) Decoding accuracy for the late delay epoch in the WM task. (c) Decoding accuracy for the cross-task decoding by training the classifier in the perceptual control task and testing it in the WM task. (d) Reconstruction fidelity values for the late delay epoch in the WM task. (e) Reconstruction fidelity values for the stimulus-presenting epoch in the perceptual control task. [file elife-94191-supp1.docx]

**Supplementary File 1**

**Supplementary File 1a.**

Decoding accuracy for the stimulus-presenting epoch in the perceptual control task

| One-sample t-test against chance (1/3) based on non-parametric permutation test | | | | | | | | |
| --- | --- | --- | --- | --- | --- | --- | --- | --- |
|  | V1 | V2 | V3 | V3AB | IPS0/1 | IPS2/3 | sPCS | iPCS |
| Within radial | ***t* = 10.433**  ***p* < .001** | ***t* = 15.598**  ***p* < .001** | ***t* = 9.802**  ***p* < .001** | ***t* = 3.848**  ***p* < .001** | ***t* = 2.049**  ***p* = 0.033** | *t* = 0.606  *p* = 0.245 | *t* = 0.712  *p* = 0.259 | *t* = 0.039  *p* = 0.510 |
| Within angular | ***t* = 8.885**  ***p* < .001** | ***t* = 6.339**  ***p* < .001** | ***t* = 7.416**  ***p* < .001** | ***t* = 3.879**  ***p* < .001** | *t* = 1.532  *p* = 0.067 | ***t* = 4.691**  ***p* < .001** | *t* = -0.218  *p* = 0.594 | *t* = 0.914  *p* = 0.172 |
| Cross radial to angular | *t* = -5.255  *p* = 1.000 | *t* = -2.993  *p* = 0.994 | *t* = -0.406  *p* = 0.621 | *t* = 1.669  *p* = 0.056 | *t* = 1.029  *p* = 0.153 | ***t* = 1.702**  ***p* = 0.049** | *t* = 1.535  *p* = 0.074 | *t* = -1.155  *p* = 0.873 |
| Cross angular to radial | *t* = -5.660  *p* = 1.000 | *t* = -3.342  *p* = 0.998 | *t* = -0.598  *p* = 0.692 | ***t* = 2.912**  ***p* = 0.008** | ***t* = 1.880**  ***p* = 0.040** | ***t* = 2.483**  ***p* = 0.009** | *t* = 0.470  *p* = 0.340 | *t* = -0.751  *p* = 0.787 |
| Permutation-based 2-way ANOVA. Decoding type $\times$ Modulator type | | | | | | | | |
|  | V1 | V2 | V3 | V3AB | IPS0/1 | IPS2/3 | sPCS | iPCS |
| Decoding type  df: (1, 15) | ***F* = 233.710**  ***p* < .001** | ***F* = 170.450**  ***p* < .001** | ***F* = 68.036**  ***p* < .001** | ***F* = 7.515**  ***p* = 0.007** | *F* = 1.145  *p* = 0.290 | *F* = 1.875  *p* = 0.179 | *F* = 0.447  *p* = 0.520 | *F* = 1.659  *p* = 0.202 |
| Modulator type  df: (1, 15) | ***F* = 6.676**  ***p* = 0.018** | ***F* = 12.017**  ***p* = 0 .001** | ***F* = 4.624**  ***p* = 0.037** | *F* < .001  *p* = 0.993 | *F* = 0.324  *p* = 0.576 | ***F* = 7.597**  ***p* = 0.005** | *F* = 0.778  *p* = 0.375 | *F* = 0.428  *p* = 0.510 |
| Decoding type  $\times$ Modulator type  df: (1, 15) | *F* = 1.251  *p* = 0.274 | ***F* = 7.585**  ***p* = 0.009** | *F* = 3.887  *p* = 0.051 | *F* = 0.632  *p* = 0.435 | *F* = 0.243  *p* = 0.621 | *F* = 2.940  *p* = 0.094 | *F* = 0.003  *p* = 0.959 | *F* = 0.035  *p* = 0.855 |

**Supplementary File 1b.**

Decoding accuracy for the late delay epoch in the WM task

| One-sample t-test against chance (1/3) based on non-parametric permutation test | | | | | | | | |
| --- | --- | --- | --- | --- | --- | --- | --- | --- |
|  | V1 | V2 | V3 | V3AB | IPS0/1 | IPS2/3 | sPCS | iPCS |
| Within radial | ***t* = 5.302**  ***p* < .001** | ***t* = 4.520**  ***p* < .001** | ***t* = 4.337**  ***p* < .001** | ***t* = 5.891**  ***p* < .001** | ***t* = 4.910**  ***p* < .001** | ***t* = 5.141**  ***p* < .001** | ***t* = 2.706**  ***p* = 0.007** | ***t* = 2.164**  ***p* = 0.025** |
| Within angular | ***t* = 5.279**  ***p* < .001** | ***t* = 6.040**  ***p* < .001** | ***t* = 4.737**  ***p* < .001** | ***t* = 6.260**  ***p* < .001** | ***t* = 5.225**  ***p* < .001** | ***t* = 3.460**  ***p* < .001** | ***t* = 2.936**  ***p* = 0.008** | *t* = 1.718  *p* = 0.058 |
| Cross radial to angular | ***t* = 4.513**  ***p* < .001** | ***t* = 4.400**  ***p* < .001** | ***t* = 4.015**  ***p* < .001** | ***t* = 4.953**  ***p* < .001** | ***t* = 4.102**  ***p* < .001** | ***t* = 3.755**  ***p* < .001** | ***t* = 1.835**  ***p* = 0.049** | *t* = 0.476  *p* = 0.354 |
| Cross angular to radial | ***t* = 4.695**  ***p* < .001** | ***t* = 4.361**  ***p* < .001** | ***t* = 3.993**  ***p* < .001** | ***t* = 5.239**  ***p* < .001** | ***t* = 4.764**  ***p* < .001** | ***t* = 3.873**  ***p* < .001** | ***t* = 4.636**  ***p* < .001** | ***t* = 2.588**  ***p* = 0.009** |
| Permutation-based 2-way ANOVA. Decoding type $\times$ Modulator type | | | | | | | | |
|  | V1 | V2 | V3 | V3AB | IPS0/1 | IPS2/3 | sPCS | iPCS |
| Decoding type  df: (1, 15) | *F* = 2.553  *p* = 0.119 | *F* = 0.931  *p* = 0.339 | *F* = 0.232  *p* = 0.646 | *F* = 0.426  *p* = 0.527 | *F* = 0.138  *p* = 0.721 | *F* = 0.012  *p* = 0.912 | *F* = 1.172  *p* = 0.290 | *F* = 0.652  *p* = 0.415 |
| Modulator type  df: (1, 15) | *F* = 0.777  *p* = 0.378 | *F* = 0.153  *p* = 0.699 | *F* = 0.205  *p* = 0.655 | *F* = 0.008  *p* = 0.926 | *F* = 0.017  *p* = 0.900 | *F* = 0.003  *p* = 0.956 | *F* = 0.624  *p* = 0.433 | *F* = 1.017  *p* = 0.325 |
| Decoding type  $\times$ Modulator type  df: (1, 15) | *F* = 1.173  *p* = 0.283 | *F* = 0.448  *p* = 0.509 | *F* = 0.161  *p* = 0.684 | *F* = 0.024  *p* = 0.876 | *F* = 0.240  *p* = 0.624 | *F* = 0.207  *p* = 0.656 | *F* = 0.092  *p* = 0.771 | *F* = 0.255  *p* = 0.613 |

**Supplementary File 1c.**

Decoding accuracy for the cross-task decoding by training the classifier in the perceptual control task and testing it in the WM task

| One-sample t-test against chance (1/3) based on non-parametric permutation test | | | | | | | | |
| --- | --- | --- | --- | --- | --- | --- | --- | --- |
|  | V1 | V2 | V3 | V3AB | IPS0/1 | IPS2/3 | sPCS | iPCS |
| Within radial | ***t* = 3.961**  ***p* < .001** | ***t* = 5.596**  ***p* < .001** | ***t* = 4.430**  ***p* < .001** | ***t* = 3.373**  ***p* < .001** | ***t* = 1.927**  ***p* = 0.031** | *t* = 1.462  *p* = 0.082 | *t* = 0.080  *p* = 0.482 | ***t* = 1.913**  ***p* = 0.042** |
| Within angular | *t* = -1.907  *p* = 0.963 | *t* = -0.213  *p* = 0.598 | *t* = 1.711  *p* = 0.050 | ***t* = 4.187**  ***p* < .001** | ***t* = 3.143**  ***p* = 0.002** | ***t* = 1.923**  ***p* = 0.036** | ***t* = 2.010**  ***p* = 0.019** | *t* = -0.183  *p* = 0.574 |
| Cross radial to angular | ***t* = 4.234**  ***p* < .001** | ***t* = 5.137**  ***p* < .001** | ***t* = 5.223**  ***p* < .001** | ***t* = 3.378**  ***p* = 0.004** | *t* = 1.282  *p* = 0.106 | ***t* = 2.752**  ***p* = 0.005** | ***t* = 2.215**  ***p* = 0.014** | *t* = 1.207  *p* = 0.132 |
| Cross angular to radial | *t* = -0.395  *p* = 0.632 | *t* = 0.444  *p* = 0.336 | ***t* = 1.821**  ***p* = 0.042** | ***t* = 3.410**  ***p* = 0.003** | ***t* = 4.227**  ***p* < .001** | ***t* = 3.652**  ***p* = 0.002** | *t* = 0.798  *p* = 0.212 | ***t* = 1.717**  ***p* = 0.049** |
| Permutation-based 2-way ANOVA. Decoding type $\times$ Modulator type | | | | | | | | |
|  | V1 | V2 | V3 | V3AB | IPS0/1 | IPS2/3 | sPCS | iPCS |
| Decoding type  df: (1, 15) | *F* = 0.848  *p* = 0.371 | *F* = 0.403  *p* = 0.523 | *F* = 0.105  *p* = 0.749 | *F* = 0.273  *p* = 0.598 | *F* = 0.011  *p* = 0.916 | *F* = 0.176  *p* = 0.691 | *F* = 0.410  *p* = 0.511 | *F* = 0.378  *p* = 0.539 |
| Modulator type  df: (1, 15) | ***F* = 33.587**  ***p* < .001** | ***F* = 30.100**  ***p* < .001** | ***F* = 6.287**  ***p* = 0.016** | *F* = 0.260  *p* = 0.612 | *F* = 2.488  *p* = 0.122 | *F* = 0.810  *p* = 0.380 | *F* = 0.026  *p* = 0.879 | *F* = 1.009  *p* = 0.312 |
| Decoding type  $\times$ Modulator type  df: (1, 15) | *F* = 0.167  *p* = 0.690 | *F* < .001  *p* = 0.975 | *F* = 0.127  *p* = 0.728 | *F* = 0.090  *p* = 0.772 | *F* = 0.993  *p* = 0.329 | *F* = 0.198  *p* = 0.660 | *F* = 3.517  *p* = 0.063 | *F* = 0.825  *p* = 0.361 |

**Supplementary File 1d.**

Reconstruction fidelity values for the late delay epoch in the WM task

| One-sample t-test against zero based on non-parametric permutation test | | | | | | | | |
| --- | --- | --- | --- | --- | --- | --- | --- | --- |
|  | V1 | V2 | V3 | V3AB | IPS0/1 | IPS2/3 | sPCS | iPCS |
| Radial | ***t* = 8.061**  ***p* < .001** | ***t* = 8.233**  ***p* < .001** | ***t* = 5.825**  ***p* < .001** | ***t* = 6.891**  ***p* < .001** | ***t* = 3.025**  ***p* = 0.004** | *t* = 0.672  *p* = 0.263 | *t* = 0.686  *p* = 0.240 | *t* = -0.359  *p* = 0.642 |
| Angular | ***t* = 10.875**  ***p* < .001** | ***t* = 11.998**  ***p* < .001** | ***t* = 9.070**  ***p* < .001** | ***t* = 7.559**  ***p* < .001** | ***t* = 3.132**  ***p* = 0.004** | *t* = 0.113  *p* = 0.492 | *t* = -1.247  *p* = 0.870 | *t* = 0.975  *p* = 0.149 |

**Supplementary File 1e.**

Reconstruction fidelity values for the stimulus-presenting epoch in the perceptual control task

| One-sample t-test against zero based on non-parametric permutation test | | | | | | | | |
| --- | --- | --- | --- | --- | --- | --- | --- | --- |
|  | V1 | V2 | V3 | V3AB | IPS0/1 | IPS2/3 | sPCS | iPCS |
| Radial | ***t* = 57.306**  ***p* < .001** | ***t* = 72.482**  ***p* < .001** | ***t* = 18.235**  ***p* < .001** | ***t* = 5.002**  ***p* < .001** | *t* = -0.183  *p* = 0.562 | *t* = 0.157  *p* = 0.436 | *t* = -0.886  *p* = 0.813 | *t* = -0.781  *p* = 0.788 |
| Angular | *t* = -4.339  *p* = 1.000 | *t* = -2.451  *p* = 0.993 | *t* = 0.059  *p* = 0.467 | ***t* = 7.945**  ***p* < .001** | ***t* = 2.013**  ***p* = 0.037** | *t* = 1.351  *p* = 0.110 | *t* = -0.864  *p* = 0.818 | *t* = -0.338  *p* = 0.644 |
